# Assessing mood and cognitive functioning in acute stroke: clinical usability of a Visual Analogue Mood Scale (VAMS)

**DOI:** 10.1007/s10072-021-05440-7

**Published:** 2021-08-08

**Authors:** Fabrizio Pasotti, Sabrina Serranò, Edoardo Nicolò Aiello, Chiara Gramegna, Matteo Querzola, Marcello Gallucci, Giuseppe Micieli, Allesandra Bollani, Elio Clemente Agostoni, Gabriella Bottini

**Affiliations:** 1 Department of Brain and Behavioural Sciences, University of Pavia, Pavia, Italy; 2 School of Medicine and Surgery, University of Milano-Bicocca, Monza, Italy; 3 PhD Program in Neuroscience, University of Milano-Bicocca, Monza, Italy; 4 Department of Psychology, University of Milano-Bicocca, Milan, Italy; 5 Cognitive Neuropsychology Centre, ASST Grande Ospedale Metropolitano Niguarda, Milan, Italy; 6 Milan Center for Neuroscience (NeuroMI), Milan, Italy; 7 Neurology Department, Fondazione Istituto Neurologico Casimiro Mondino, Pavia, Italy; 8 Neurology and Stroke Unit, ASST Grande Ospedale Metropolitano Niguarda, Milan, Italy

**Keywords:** Depression, Acute stroke, Stroke, visual analogue scale, Mood, Cognitive functioning

## Abstract

**Background:**

Patients suffering from stroke in the acute/post*-*acute phases often present with depressive mood — which negatively impacts on patients’ prognosis. However, psychometric evaluation of mood in acute stroke patients may be challenging due to cognitive deficits. Tools investigating emotional states via a vertical analogue line may overcome language/visuo-spatial disorders. This study thus aimed at (a) investigating the clinical usability of a Visual Analogue Mood Scale (VAMS) in acute stroke patients and (b) investigating the interplay between mood and cognition in this population.

**Methods:**

Forty-one acute stroke patients were compared to 41 age-, education- and sex-matched healthy participants (HPs) on the VAMS and on cognitive measures (mental performance in acute stroke, MEPS). A control line bisection (LB) task was administered to control for potential visuo-spatial deficits in patients.

**Results:**

Patients reported higher depression levels than HPs (lower VAMS scores); this between-group difference stayed significant when covarying for LB scores. MEPS scores discriminated patients from HPs; among cognitive measures, only the Clock drawing test (CDT) was positively associated with VAMS scores. Lesion side did not affect patients’ mood state; however, disease duration was inversely related to VAMS scores.

**Discussion:**

The VAMS proved to be a suitable tool for assessing mood in acute stroke patients, as being independent from post-stroke cognitive sequelae. The CDT might represent an adequate measure of depression-induced, post*-*stroke cognitive efficiency decrease. Mood disorders might occur and thus should be adequately addressed also in post-acute phases — likely due to longer hospitalization times and regression of anosognosic features.

## Introduction

A stroke occurs when the blood flow directed to the brain is suddenly interrupted by embolic, ischemic or hemorrhagic events, leading to brain damage. Cerebral ischemia is reckoned to be the most frequent cause of stroke (87%) [28].

According to the World Health Organization, 15 million people suffer from stroke worldwide each year [29].

Between 2015 and 2018, stroke incidence in the USA has been estimated at 7.6 million (≥ 20 years), with an expected increase of 3.4 million by the year 2030 (≥ 18 years) when compared to the year 2012 [28]. Advanced age being one of the main risk factors, its exponential growth may determine a 50% increase in the death toll by 2030 when compared to 2012 [28].

Stroke is also one of the leading causes of death and long-term disability [28].

Among both short- and long-term functional sequelae, a stroke can cause several cognitive and mood disorders — depending on brain structures affected, as well as on the features of the lesion [6, 22, 23].

Neglect, aphasia, apraxia and dysexecutive symptoms are highly frequent neuropsychological consequences [5, 6, 9], whereas depression may arise within the mood sphere [21–23] and mood symptoms in general are considered a potentially direct consequence of stroke [21, 22].

As mood and cognition dysfunctions represent negative prognostic factors for both recovery [5, 11, 24] and survival [16], it is crucial to provide neuropsychological assessment, even at an early stage [25].

Typically, in the acute stage of stroke, patients are bedridden and fatigued and show fluctuating levels of arousal and awareness [8]. Therefore, short-lived although specific screening tools appear to be more reliable [25].

Given the frequent association between depression and cognitive dysfunctions in stroke [12, 22] the present study aimed at the following: (a) exploring the interplay between mood and cognition in a clinic-based cohort of patients in the acute post-stroke phase; (b) investigating the clinical usability of a mood scale that accounts for cognitive dysfunctions [1, 2].

## Methods

### Participants

A convenience cohort of forty-one stroke patients was consecutively recruited at Stroke Units/Neurology Sections of four hospitals in Northern Italy: ASST Grande Ospedale Metropolitano Niguarda (Milan); Fondazione Istituto Neurologico Casimiro Mondino (Pavia); Ospedale Civile di Voghera; Humanitas Clinical and Research Hospital (Rozzano). Inclusion criteria were (a) ischemic or hemorrhagic stroke supported by cerebral computerized tomography/magnetic resonance imaging; (b) acute phase (<30 days from the event); (c) age >18 years. Patients had no history of psychiatric or other neurological co-morbidities, drug abuse, and multi-organ impairments. Severe aphasia with impaired comprehension (e.g., global aphasia) and/or severe disorders of consciousness were addressed as further exclusion criteria — as preventing from assessing patients cognition and mood.

The control group consisted of 41 age-, education- and sex-matched healthy participants (HPs) — i.e., with no history of neurological, psychiatric or severe internal conditions. The Mini-Mental State Examination [14, 15] was administered to HPs to rule out cognitive impairment.

Participants were native Italian speakers and had normal or corrected-to-normal vision.

Participants provided written informed consent before being enrolled. The study was approved by the local Research Ethics Committee and conducted according to the Declaration of Helsinki.

### Materials

#### Cognitive assessment

Cognition was assessed via the mental performance in acute stroke (MEPS) [18], a cognitive screener developed to assess both instrumental and non-instrumental domains in patients suffering from stroke in the acute stages. The MEPS encompasses a set of 14 both verbal and non-verbal tasks evaluating orientation (temporal, TO; spatial, SO), language (Order comprehension, OC; Reading and comprehension of sentence, RCS; Words repetition, WR; Picture naming, PN), memory (Digit span, DS; Immediate visual memory, IVM), praxis (Ideomotor apraxia, IdeomA; Ideative apraxia, IdeatA) and visuo-spatial abilities (Segments discrimination, SD; Visual exploration and attention, VEA; Clock drawing test, CDT) and abstraction (Similarity judgments, SJ).

#### Mood assessment

Mood was assessed via a visual analogue scale (Visual Analogue Mood Scale; VAMS) [1, 2, 27]. The VAMS consists of a 180-mm line having two circles at its respective ends that represent two opposite moods — the upper circle containing a stylized “happy” face, the lower a “sad” one. The VAMS thus allows measuring euthymia/dysthymia along a *continuum*.

As being a non-verbal, self-reported and rapid tool, it can be indicated for people with language difficulties — such as post-stroke patients [6]. Moreover, to prevent from any potential stroke-related attentional/representational/visuo-spatial deficit affecting patients’ performance, VAMS stimuli were conveniently structured vertically [27].

Participants were asked to draw a mark with the pen at the point that corresponded to his/her mood at that specific moment.

To ensure reliability in responses, the mean of 3 consecutively administered measures was regarded as the outcome.

#### Line bisection task

In order to control for systematic biases in VAMS scores due to potential attentional/representational/visuo-spatial deficits (e.g., neglect), a vertical line-bisection (LB) task was administered. The LB task consisted of a 180-mm-long vertical straight line having two empty circles at their respective ends (one for each end). The subject was asked to mark with a pen the middle of the line. To ensure reliability in responses, the mean of 3 consecutively administered measures was regarded as the outcome.

### Statistical analyses

Normality checks were performed on raw variables both descriptively (by assessing skewness and kurtosis values, judged as abnormal if > |1| and |3|, respectively) and graphically (by visually inspecting histograms and Q-Q plots) [7, 13]. Associations of interest between continuous variables were assessed via either Pearson’s or Spearman’s techniques; between-group comparisons were explored via either Mann-Whitney and Kruskal-Wallis tests or *t*-tests and analyses of variance.

Clinical judgments on MEPS total and sub-test scores were drawn according to the Equivalent Scores (ESs) method [3, 18].

Significance level was set at .05. Analyses were performed via SPSS 27 [10].

## Results

Background, clinical and psychometric measures of participants are summarized in Table 1. Groups were matched for age (*t*(59.02)=.16; *p*=.874) and education (*t*(80)=−.45; *p*=.651). Male/female ratio was balanced in each group (χ^2^(1)=.05; *p*=.822). Global cognition (MEPS total score) was not influenced by lesion side (χ^2^(2)=3.2; *p*=.205).

**Table 1:** Participants’ background, clinical and psychometric features

	*N*	Sex (F/M)	Age (years)	Education (years)	Days from onset	Lesion side (L/R/B)	VAMS	LB	MEPS total
Patients	41	24/17	64.2 ± 11.4 (45–90)	9.2 ± 4 (1–18)	4.2 ± 2.6 (1–10)	16/17/8	105.2 ± 54.2 (5.3–174)	92.3 ± 6.4 (78.3–105.7)	73.3 ± 6.8 (54–81.8)
HPs	41	25/16	63.9 ± 5.7 (55–76)	9.6 ± 3.3 (5–13)	–	–	128.1 ± 36.7 (25.7–173.3)	89 ± 3.2 (82.3–94.3)	79.8 ± 1.8 (74.4–82)

Notes: *HPs*, healthy participants; *F*, female; *M*, male; *L*, left; *R*, right; *B*, bilateral; *VAMS*, Visual Analogue Mood Scale; *LB*, line bisection task; *MEPS*, mental performance in acute stroke

HPs performed better than patients on both MEPS total (*z*=−5.61; *p*<.001) and sub-tests scores — TO (*z*=−4.73; *p*<.001), SO (*z*=−3.9; *p*<.001), OC (*z*=−2.53; *p*=.012), SD (*z*=−3.8; *p*<.001), RCS (*z*=−2.96; *p*=.003), IVM (*z*=−2.97; *p*=.003), WR (*z*=−2.04; *p*=.042), CDT (*z*=−3.65; *p*<.001), SJ (*z*=−4.73; *p*<.001), and PN (*z*=−3.35; *p*=.001). No significant between-group differences were detected with regard to DS, VEA, IdeomA and IdeatA. 51.2% of patients (*N*=21) scored below the cut-off on total-MEPS.

Neither in patients nor in HPs, sex, age or education was found to be associated with VAMS scores. When compared to HPs, patients reported lower VAMS (*t*(70.26)=−2.24, *p*=.029) and higher LB (*t*(58.84)=3.02, *p*=.004) scores. The between-group difference in VAMS scores stayed significant even when covarying for LB scores (*F*(1,79)=7.27; *p*=.009).

The association between VAMS scores and cognitive measures possibly affecting the execution of the task (i.e., MEPS total, SD, RCS, AM, CDT) was tested in both groups. Patients’ VAMS scores proved to be positively related to the performance on the CDT (*r*_*s*_(41)=.34; *p*=.027), whereas no other significant associations were detected.

No differences were found in patients’ VAMS scores between those who performed below (ES=0) and above (ES≥1) total-MEPS (*t*(39)=−1.58; *p*=.123) and CDT (*t*(39)=−.65; *p*=.523) cut-off values.

Patients who scored above 90 (“euthymic”) at the VAMS had been hospitalized for a shorter period of time (*M*=3.27; *SD*=1.5) when compared (*t*(17.24)=17.24; *p*=.008) to those who scored below 90 (“dysthymic”; *M*=5.9; *SD*=3.3). Moreover, a negative association was found between days from onset and VAMS scores (*r*(39)=−.52; *p*=.001).

## Discussion

The VAMS appears to be a feasible tool for assessing emotional status (sadness) in patients with stroke independently of specific attentional/representational/visuo-spatial deficits. The correlation between the CDT and VAMS scores might be due to a post-stroke global cognitive dysfunction [19].

Our results also support the hypothesis that the occurrence of depressive symptoms is not lesion-side specific [17, 22].

In addition, the correlation between the CDT and VAMS scores suggests that the CDT is an adequate task for assessing cognition in acute/post-acute stroke patients with mood disorders [20].

An inverse correlation between VAMS scores and the number of days from stroke onset was also found, suggesting that the increasing awareness of the disease [26], together with the burden due to the prolonged hospitalization, negatively influences patients’ mood. This finding further supports the relevance of mood assessment also in post-acute stroke patients.

The present work is not free of limitations.

First, the VAMS was not put into relation with other quantitative/qualitative measures of depressive symptoms — this preventing from assessing its convergent validity as well as its sensitivity and specificity. Thereupon, further studies are undoubtedly needed in order to provide an in-depth analysis of VAMS both psychometric and diagnostic properties. However, it has to be noted that even gold-standard mood scales might be inefficient in detecting mood disorders in this population [4]. Similarly, should the VAMS be compared to clinical judgments, this comparison might suffer from subjectivity in ratings — which would increase due to intervening influences of the acute phase.

Moreover, it is worth noting that patients were not classified according to post-stroke cognitive syndromic nosography (e.g., aphasia, neglect). However, the fact that lesion side did not affect mood levels might, at least to an extent, rule out the influence of domain-specific cognitive impairment on VAMS performances.
